# Efficacy and safety of chemotherapy after endoscopic double stenting for malignant duodenal and biliary obstructions in patients with advanced pancreatic cancer: a single-institution retrospective analysis

**DOI:** 10.1186/s12876-018-0886-8

**Published:** 2018-10-26

**Authors:** Kazuyuki Matsumoto, Hironari Kato, Shigeru Horiguchi, Koichiro Tsutsumi, Yosuke Saragai, Saimon Takada, Sho Mizukawa, Shinichiro Muro, Daisuke Uchida, Takeshi Tomoda, Hiroyuki Okada

**Affiliations:** Department of Gastroenterology and Hepatology, Okayama University Graduate School of Medicine, Dentistry, and Pharmaceutical Science, 2-5-1 Shikata-cho, Okayama, 700-8558 Japan

**Keywords:** Chemotherapy, Double stenting, Pancreatic cancer

## Abstract

**Background:**

Advanced pancreatic cancer is accompanied not only by bile duct obstruction, but also occasionally by duodenal obstruction. With new advances in chemotherapy and improvement in the management of stent dysfunction, the life expectancy of patients with pancreatic cancer has increased. This study aimed to evaluate the efficacy and safety of chemotherapy for advanced pancreatic cancer, as well as to analyze the prognostic factors, following endoscopic double stenting.

**Methods:**

This retrospective study was conducted from January 1, 2007 to October 31, 2015 at an academic center. Fifty consecutive patients with pancreatic cancer who had undergone endoscopic double stenting, comprising duodenal and biliary stenting, were analyzed. We reviewed the patients records and analyzed the data of stent dysfunction rates after double stenting, reintervention for stent dysfunction, chemotherapy after double stenting, adverse events associated with chemotherapy after double stenting, survival times following double stenting, and overall survival times. The hospital’s institutional review board for human research approved this study.

**Results:**

The overall survival time and the survival time following double stenting were 10.9 months (IQR 6.0–18.4 months) and 2.4 months (IQR 1.4–5.2 months), respectively. After double stenting, duodenal stent dysfunction occurred in 6 patients (12%), and biliary stent dysfunction occurred in 12 patients (24%), respectively. All patients who experienced stent dysfunction underwent endoscopic reintervention, and all of the procedures were successful. Twenty-one (42%) patients were treated with chemotherapy post double stenting; 9 patients received chemotherapy as a first-line treatment, 9 as a second-line treatment, and 3 as a third-line treatment. During chemotherapy, 8 (38%) patients had grade 3–4 adverse events, which were manageable. Chemotherapy post double stenting (OR, 0.19; 95% CI, 0.059–0.60; *P* = .0051), reintervention for biliary stent dysfunction (OR, 0.21; 95% CI, 0.081–0.50; *P* = .0002), and performance status (< 2) (OR, 0.28; 95% CI, 0.098–0.71; *P* = .0064) were significant prognostic factors after double stenting.

**Conclusions:**

Systemic chemotherapy was manageable, even in patients with double stenting. Chemotherapy after double stenting and appropriate reintervention for stent obstructions potentially prolonged the survival of patients with advance pancreatic cancer.

## Background

Pancreatic cancer is the fourth leading cause of cancer-related death in the United States [1] and fifth in Japan. Although surgical resection is the only curative treatment for this disease, more than 80% of patients with pancreatic cancer are diagnosed at unresectable stage. Advanced pancreatic cancer is accompanied not only by bile duct obstruction, but also sometimes by duodenal obstruction [2, 3]. These cause symptoms such as nausea, vomiting, anorexia, and weight loss, resulting in a marked decline in quality of life. Recently, the efficacy of treatment using biliary stenting (BS), which includes endoscopic retrograde cholangiopancreatography biliary drainage (ERCP-BD) and endoscopic ultrasound-guided (EUS)-BD, and duodenal stenting (DuS; also known as double stenting), has been described to improve the clinical symptoms of patients [4–9].

With the advent of new oncologic therapies and improvement in the management of adverse events of chemotherapy, the life expectancy of patients with pancreatic cancer has steadily increased [10–12]. In a recent study, patients who received chemotherapy after placement of duodenal stent for advanced cancer show better survival than the patients who did not receive chemotherapy [13–15]. Control of clinical symptoms associated with advanced cancer, such as gastric outlet obstruction (GOO) and obstructive jaundice, is required.

Systemic chemotherapy in patients who had undergone double stenting for advanced pancreatic cancer is difficult, because management of both bile duct and duodenal stents are required. Current studies showed that endoscopic double stenting, including reintervention for stent dysfunction, is technically feasible and clinically effective for advanced pancreatobiliary cancer [5, 9]. However, chemotherapy in patients who had undergone double stenting has not been reported. Therefore, in this study, we aimed to evaluate the efficacy and safety of chemotherapy for advanced pancreatic cancer, as well as to analyze the prognostic factors, following double stenting.

## Methods

### Patients

Fifty consecutive patients with pancreatic cancer who had undergone endoscopic double stenting, comprising DuS and BS, from January 1, 2007 to October 31, 2015 at our institution, were retrospectively analyzed. The records of these patients were reviewed, and the following data were analyzed: patient characteristics, duodenal stenosis sites, timings of double stenting, selected BD methods, stent patency periods, technical and clinical success rates for reintervention, chemotherapy after double stenting, adverse events associated with chemotherapy after double stenting, time from initial diagnosis to double stenting, survival times following double stenting, and overall survival times. The hospital’s institutional review board for human research approved this study.

The duodenal stenosis types were classified according to the location of the stenosis relative to the ampulla of Vater, as follows: type I: proximal to and no involvement of the ampulla of Vater; type II: affecting the second part of the duodenum and the ampulla of Vater; and type III: affecting the third part of the duodenum without ampulla of Vater involvement [5]. In simultaneous type II stenosis cases, if passage of the scope was possible, we deployed the biliary metal stent (MS) or plastic stent (PS) through ERCP-BD, followed by the DuS. If passage of the scope was impossible, we evaluated the duodenal bulb invasion of the tumor to determine whether EUS-choledochoduodenostomy (EUS-CDS) was possible. If possible, we performed EUS-CDS first, followed by the DuS. Otherwise, we selected EUS-hepaticogastrostomy (EUS-HGS) for biliary drainage. In cases of biliary stenosis after DuS for metachronous type II stenosis, we attempted a transpapillary approach through the MS mesh following insertion of the scope into the lumen of the duodenal stent. If the transpapillary approach failed, we performed EUS-BD. EUS-CDS was adopted for cases without duodenal bulb invasion, while EUS-HGS was adopted for duodenal bulb invasion.

The method of reintervention for ERCP-BDs was following, a duodenal scope was inserted through the duodenal stent. PS exchange involved placing a guidewire into the target branch before PS removal, then inserting a 7–10 Fr PS. When tumor ingrowth or overgrowth obstructed the MS, we inserted a 5–7 Fr PS into the lumen of the previously deployed MS. The methods of reintervention for EUS-CDS was following, a duodenal scope or a forward-viewing endoscope was used. PS exchange involved occluded stent removal. Then, a seeking guidewire was passed through the fistula and a new PS was placed. When the MS obstructed with sludge, a stone retrieval balloon was used and removed the sludge.

The stent patency periods for DuS and BS were measured from the day on which double stenting was undertaken to the day on which the stent became dysfunctional, the day the patient died, or the day of the last follow-up appointment. Stent dysfunction included stent obstruction, stent migration, and cholangitis. Obstructive jaundice recurrence, which was based on laboratory examinations and biliary dilation on computed tomography (CT) images, was considered to result from biliary stent obstruction. Symptom recurrence associated with gastroduodenal obstruction, including nausea, vomiting, and difficulties with oral intakes, and a grossly dilated stomach on CT images was considered to be caused by duodenal stent obstruction. Cholangitis was defined as elevations of liver enzyme level and the presence of typical symptoms, including fever. For reintervention, clinical success of biliary stent was defined as a decrease in bilirubin level to < 75% of the pre-drainage levels within 30 days. Clinical success of duodenal stent was based on oral intakes before and after stent placement using the Gastric Outlet Obstruction Scoring System (GOOSS) [16]. Overall survival time was defined as the period between pathological diagnosis and patient death or lost to follow-up.

The basic chemotherapy regimen after double stenting is as follows: gemcitabine (Gemzar, intravenous 1000 mg/m^2^ on days 1, 8, and 15 on a 28-day cycle; Eli Lilly and Company, Indianapolis, IN, USA), S-1 (TS-1, 80–100 mg/day per oral from days 1 to 14 on a 21-day cycle; Taiho Pharmaceutical Co., Ltd., Tokyo, Japan), GEM/S-1 (Gemzar, 1000 mg/m^2^ on days 1 and 8; TS-1, 80–100 mg/day from days 1 to 14 on a 21-day cycle), irinotecan (Topotecin, intravenous 180 mg/m^2^ on a 14-day cycle; Daiichi-Sankyo Co., Ltd., Tokyo, Japan), and modified FOLFIRINOX [mFOLFIRINOX; oxaliplatin 85 mg/m^2^ (Elplat; Yakult Honsha Co., Ltd., Tokyo, Japan), irinotecan 150 mg/m^2^, leucovorin 400 mg/m^2^ (Isovorin injection; Pfizer Inc., New York, NY, USA), and continuous infusion of fluorouracil 2400 mg/m^2^ over 46 h (5-FU injection; Kyowa Hakko Kirin Co., Ltd., Tokyo, Japan), with routine subcutaneous infusion port on a 14-day cycle]. The toxicities were assessed using the National Cancer Institute Common Terminology Criteria for Adverse Events, version 4 (CTCAE v4). Chemotherapy was continued until disease progression, intolerable adverse events, or patient refusal.

### Statistical analysis

Continuous variables were expressed as median and range or interquartile range (IQR) or mean ± standard deviation (SD). Chi-square test was performed to analyze categorical variables. Student’s t-test was used to compare continuous variables. The biliary stent patency periods were estimated using the Kaplan-Meier method, and they were compared using the log-rank test. Factors with *P* values < .05 were considered survival factors after double stenting using the forward method, and they were analyzed in a multiple logistic regression model. Odds ratios (ORs) and 95% confidence intervals (CIs) were calculated. Two-tailed *P* values < .05 were considered statistically significant. All analyses were performed using JMP Pro 12 (SAS Institute, Cary, NC, USA).

## Results

### Patient characteristics

Table 1 summarizes patient characteristics. The median age of the patients was 65 years (range 41–86 years). None of the patients had previous treatment. Thirty-three (66%) patients had distant metastasis at the initial diagnosis. The median time from initial diagnosis to double stenting was 5.9 months (IQR 1.1–12.1 months). Double stenting was performed simultaneously in 21 (42%) and metachronously in 29 (58%) patients. Type I, II, and III duodenal stenosis had occurred in 20 (40%), 21 (42%), and 9 (18%) patients, respectively. GOOSS score increased from 0.9 [standard deviation (SD) 0.8] to 2.8 (SD 0.4) points following DuS (*P* < .001). Selected BD methods for double stenting were ERCP-BD in 35 (70%) patients, EUS-CDS in 12 (24%) patients, and EUS-HGS in 3 (6%) patients, respectively. The types of biliary stent used were PS in 11 (22%) patients and MS in 39 (78%) patients. Twenty-one (42%) patients were treated with chemotherapy post double stenting. The performance status at double stenting was 0–1 in 28 (56%) patients and 2–3 in 22 (44%) patients. The overall survival time and the survival time following double stenting were 10.9 months (IQR 6.0–18.4 months) and 2.4 months (IQR 1.4–5.2 months), respectively. During follow-up study, 47 (94%) patients died.

**Table 1:** Clinical characteristics and outcome of the patients who underwent double stenting

Parameter	Number
Age, median (range), years	65 (41–86)
Sex, male, *n* (%)	33 (66)
Distant metastasis at initical diagnosis, *n* (%)	33 (66)
Time from initial diagnosis to double stenting (IQR), mo	5.9 (1.1–12.1)
Timing of duodenal and biliary stenoses, *n* (%)	
Simultaneous	21 (42)
Metachronous	29 (58)
Type of duodenal stenosis, *n* (%)	
I	20 (40)
II	21 (42)
III	9 (18)
GOOSS score, mean ± SD	
Before DuS	0.9 ± 0.8
After DuS	2.8 ± 0.4
Biliary drainage methods, *n* (%)	
ERCP-BD	35 (70)
EUS-CDS	12 (24)
EUS-HGS	3 (6)
Type of biliary stent	
PS	11 (22)
MS	39 (78)
Chemotherapy post double stenting, *n* (%)	21 (42)
Performance status at the double stenting, *n* (%)	
0–1	28 (56)
2–3	22 (44)
Overall survital time (IQR), mo	10.9 (6.0–18.4)
Survival time from double stenting (IQR), mo	2.4 (1.4–5.2)

*IQR* interquartile range, *GOOSS* gastric outlet obstruction scoring system

*SD* standard deviation, *DuS* duodenal stenting

*ERCP-BD* endoscopic retrograde cholangiopancreatography-guided biliary drainage

*EUS-CDS* endoscopic ultrasound-guided choledochoduodenostomy

*EUS-HGS* endoscopic ultrasound-guided hepaticogastrostomy

*PS* plastic stent, *MS* metal stent

### Stent dysfunction after double stenting

The mean duodenal stent patency after double stenting was 106 ± 136 days (median not reached). During follow-up (median 73 days, IQR 44–131 days), duodenal stent dysfunction occurred in 6 patients (12%). All patients who experienced stent dysfunction underwent endoscopic reintervention, and all of the procedures (2 patients for overgrowth, 2 patients for food impaction, 1 patient for stent migration) were successful. The GOOSS score increased from 0.6 (SD 0.5) to 2.2 (SD 0.8) points following reintervention (*P* < .001).

The median biliary stent patency period after double stenting was 230 days (range 17–375 days). During follow-up (median 54 days, IQR 43–118 days), biliary stent dysfunction occurred in 12 patients (24%). The median biliary stent patency period for MS and PS was 270 days (range 17–337) and 81 days (19–375), respectively (*P* < .005). Of the 12 patients who experienced stent dysfunction (6 patients with MS, 6 patients with PS), 6 had undergone ERCP-BD and 6 had EUS-CDS. The median time of stent dysfunction after double stenting was 87 days (range 25–270 days). All patients who experienced stent dysfunction underwent endoscopic reintervention, and all of the procedures (7 patients for sludge, 3 patients for ingrowth, 1 patient for migration, 1 patient for kinking) were successful. However, one patient experienced clinical failure following ERCP-BD, thus the patient underwent an additional intervention using EUS-HGS. Therefore the clinical success rate was 92% (11/12).

### Post double stenting chemotherapy and adverse event

**Fig. 1:**
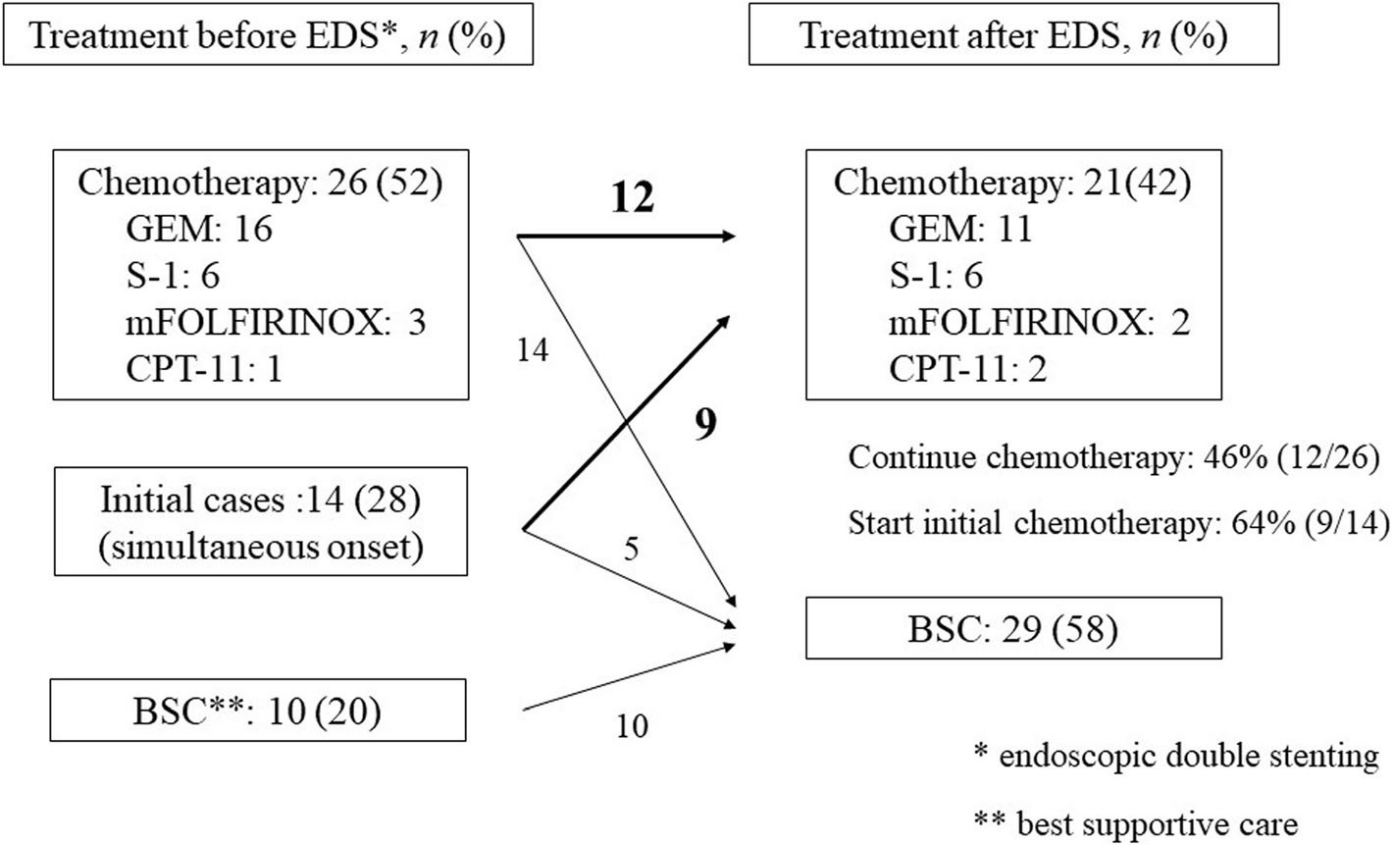
Treatment contents before and after double stenting. After double stenting, 12 (46%) out of 26 patients with chemotherapy could continue another chemotherapy, and 9 (64%) out of 14 patients with simultaneous onset could start initial chemotherapy

Prior to double stenting, 26 (52%) patients received chemotherapy and 10 (20%) patients were treated with best supportive care (BSC). Both bile duct and duodenal obstruction developed simultaneously in the remaining 14 (28%) patients at the initial diagnosis. After double stenting, 12 (46%) out of 26 patients with chemotherapy could continue another chemotherapy, and 9 (64%) out of 14 patients with simultaneous onset could start initial chemotherapy (Fig. 1). Results showed that 21 (42%) patients received chemotherapy post double stenting; 9 patients received chemotherapy as a first-line treatment, 9 as a second-line treatment, and 3 as a third-line treatment. The regimens were GEM in 8 patients, S-1 in 8 patients, CPT-11 in 2 patients, mFORFIRINOX in 2 patients, and GEM+S-1 in one patient. During chemotherapy, 8 (38%) patients had grade 3–4 adverse events: neutropenia in 4, appetite loss in 2, and diarrhea in 2. However, these adverse events were manageable by dose reduction, drug holiday, or temporary drip. No fatal adverse events associated with chemotherapy were noted.

On the other hand, 29 (58%) out of 50 patients who underwent double stenting received BSC because of contraindications to systemic chemotherapy. These contraindications include poor general condition in 22, patient refusal in 5, and refractory to the standard treatment (GEM, TS-1, or mFOLFIRINOX) in 2 patients.

### Prognostic factors after double stenting

The stent type, BD method, reintervention for duodenal stent dysfunction, reintervention for biliary stent dysfunction, chemotherapy post double stenting, and performance status were potential prognostic factors after double stenting (*P* < .05) in the univariate analyses. Multiple logistic regression determined that reintervention for biliary stent dysfunction (OR, 0.21; 95% CI, 0.081–0.50; *P* = .0002), chemotherapy post double stenting (OR, 0.19; 95% CI, 0.059–0.60; *P* = .0051), and performance status (< 2) (OR, 0.28; 95% CI, 0.098–0.71; *P* = .0064) were significant prognostic factors after double stenting (Table 2).

**Table 2:** Logistic regression analysis of survial time after double stenting

	Univariable analysis		Multivariable analysis	
Variable	OR (95% CI)	*P* value	OR (95% CI)	*P* value
Age (≥65)	0.79 (0.43–1.42)	0.43		
Gender (M)	0.77 (0.40–1.53)	0.45		
Distant metastaisis at initial diagnosis (yes)	1.26 (0.69–2.42)	0.46		
Timing of double stenting (Simultaneous)	0.64 (0.34–1.17)	0.15		
Type of stent (PS)	0.43 (0.19–0.87)	0.018	0.82 (0.29–2.05)	0.68
Biliary drainage method (EUS-BD)	0.29 (0.13–0.57)	0.0002	0.45 (0.16–1.13)	0.093
Reintervention for DU stent dysfunction (yes)	0.24 (0.078–0.59)	0.0012	0.42 (0.098–1.54)	0.19
Reintervention for BS dysfunction (yes)	0.33 (0.15–0.68)	0.0018	0.21 (0.081–0.50)	0.0002
Chemotherapy post double stenting (yes)	0.14 (0.067–0.30)	<.0001	0.19 (0.059–0.60)	0.0051
Performance status (< 2)	0.11 (0.048–0.23)	<.0001	0.28 (0.098–0.71)	0.0064

*OR* odds ratio, *CI* confidence interval, *PS* plastic stent, *EUS-BD* endoscopic ultrasound-guided biliary drainage, *DU* duodenal stent, *BS* biliary stent

### Comparison of survival times and prognostic factors

**Fig. 2:**
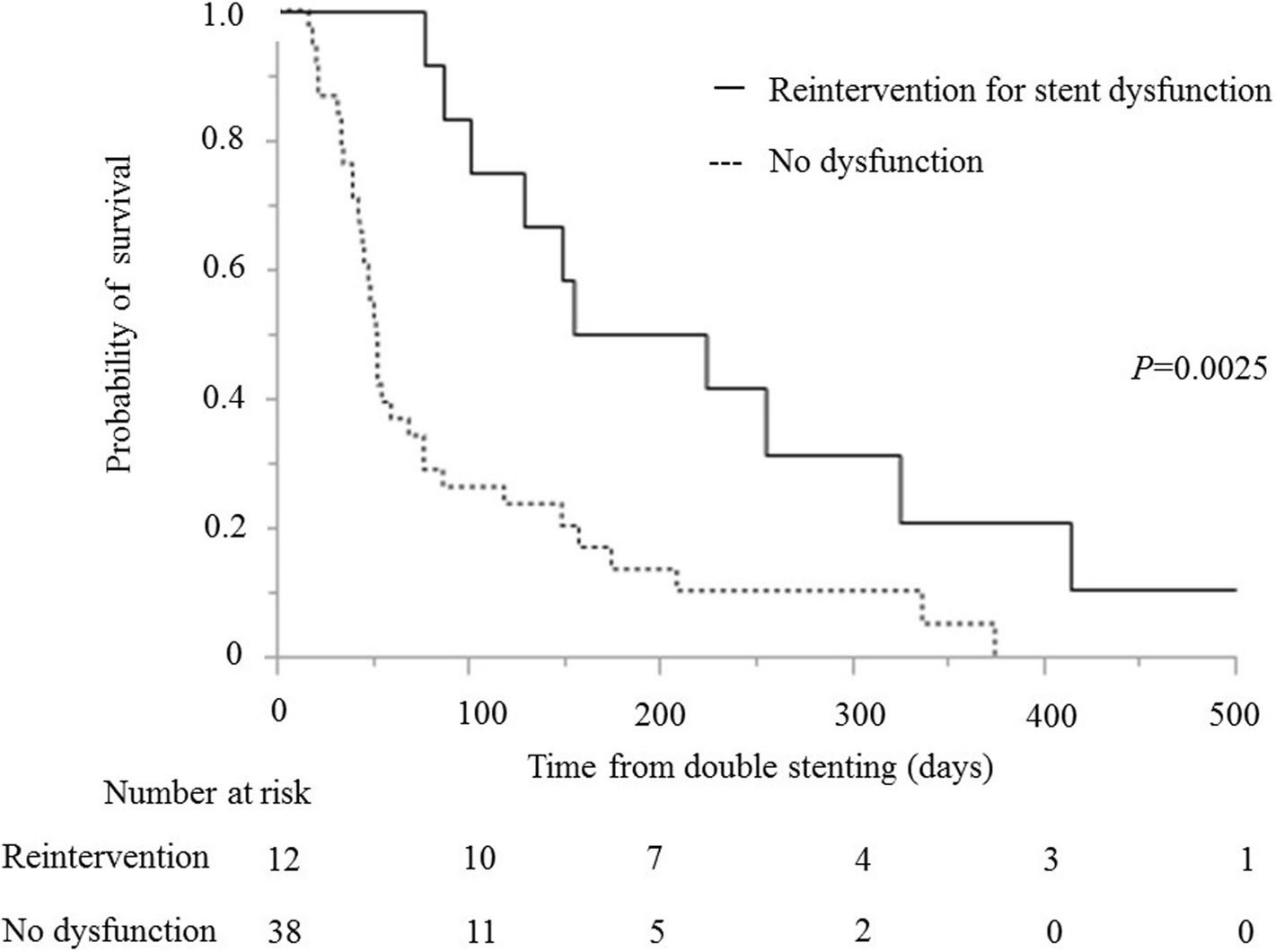
Kaplan-Meier curve showing survival time following double stenting. Reintervention for biliary stent dysfunction versus no dysfunction (189 vs 52 days; *P* = 0.0025)

**Fig. 3:**
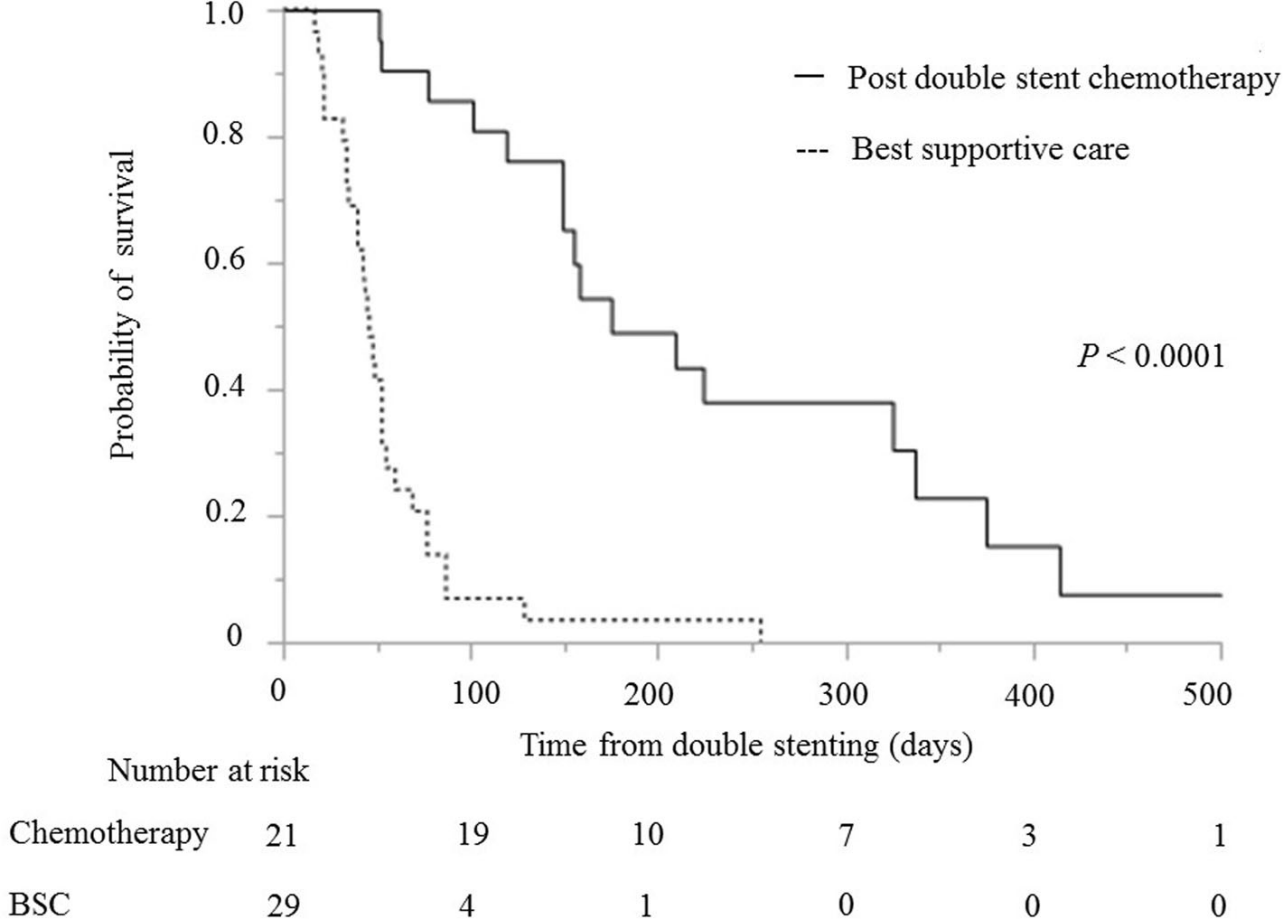
Kaplan-Meier curve showing survival time following double stenting. Chemotherapy versus best supportive care (no chemotherapy) (175 vs 46 days; *P* = <.0001)

**Fig. 4:**
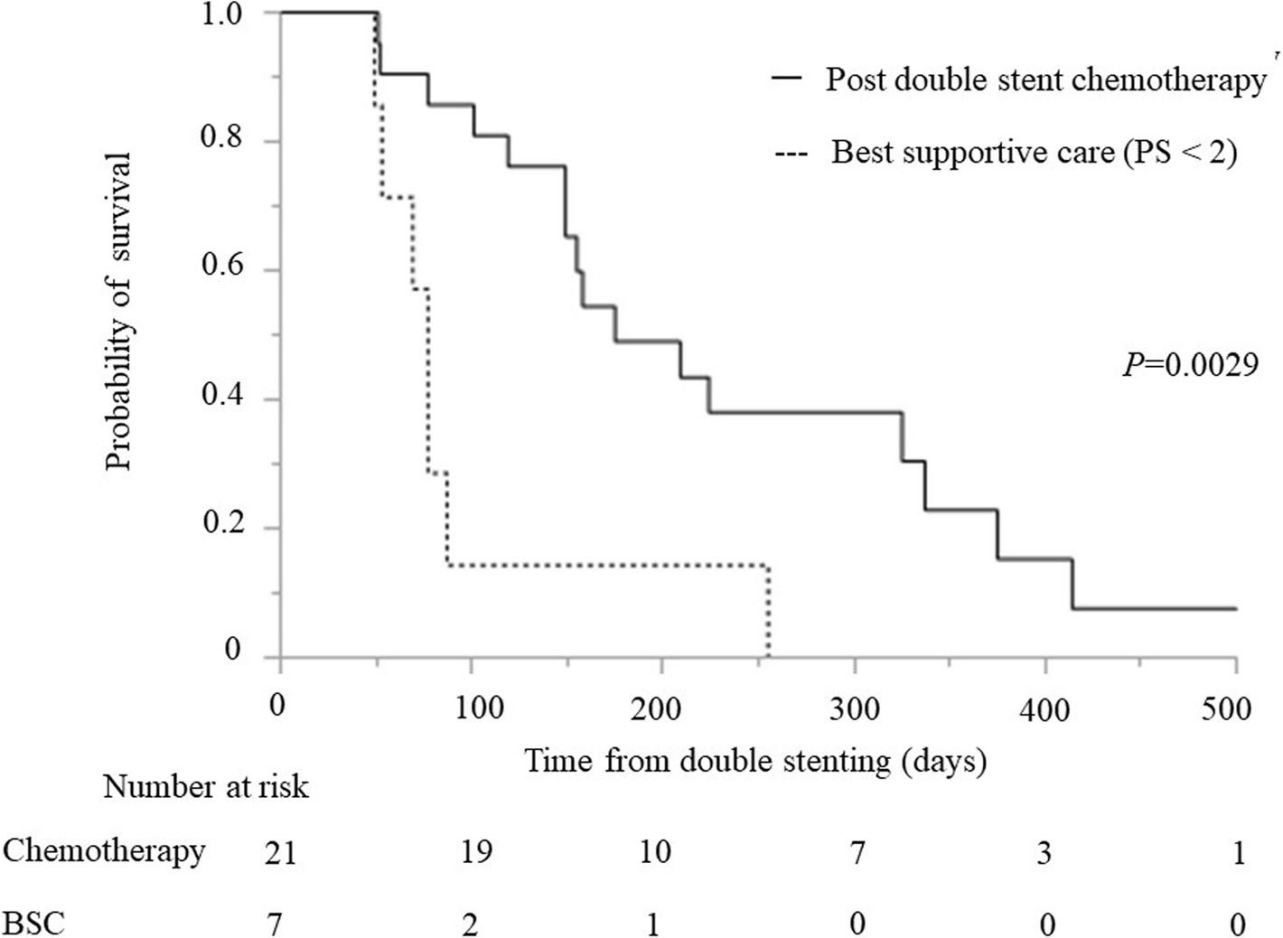
Kaplan-Meier curve showing survival time following double stenting. Chemotherapy versus best supportive care with good performance status (0 or 1) (no chemotherapy) (175 vs 77 days; *P* = 0.0029)

The median survival time after double stenting was significantly longer in patients with reintervention for BS (189 days: IQR 115–325) than that in patients without reintervention (52 days: IQR 40–119) (*P* = 0.0025) (Fig. 2). It was also longer in patients with PS <  2 (155 days: IQR 77–325) than that in patients with PS ≥ 2 (42 days: IQR 32–53) (*P* = <.0001). Moreover, the median survival time was longer in patients with chemotherapy post double stenting (175 days: IQR 149–337) than that in patients without chemotherapy (46 days: IQR 34–60) (*P* = <.0001) (Fig. 3). Considering the patients with PS <  2, the median survival time after double stenting was significantly longer in patients with chemotherapy post double stenting (175 days: IQR 149–337) than that in patients without chemotherapy (77 days: IQR 53–87) (*P* = 0.0029) (Fig. 4).

**Fig. 5:**
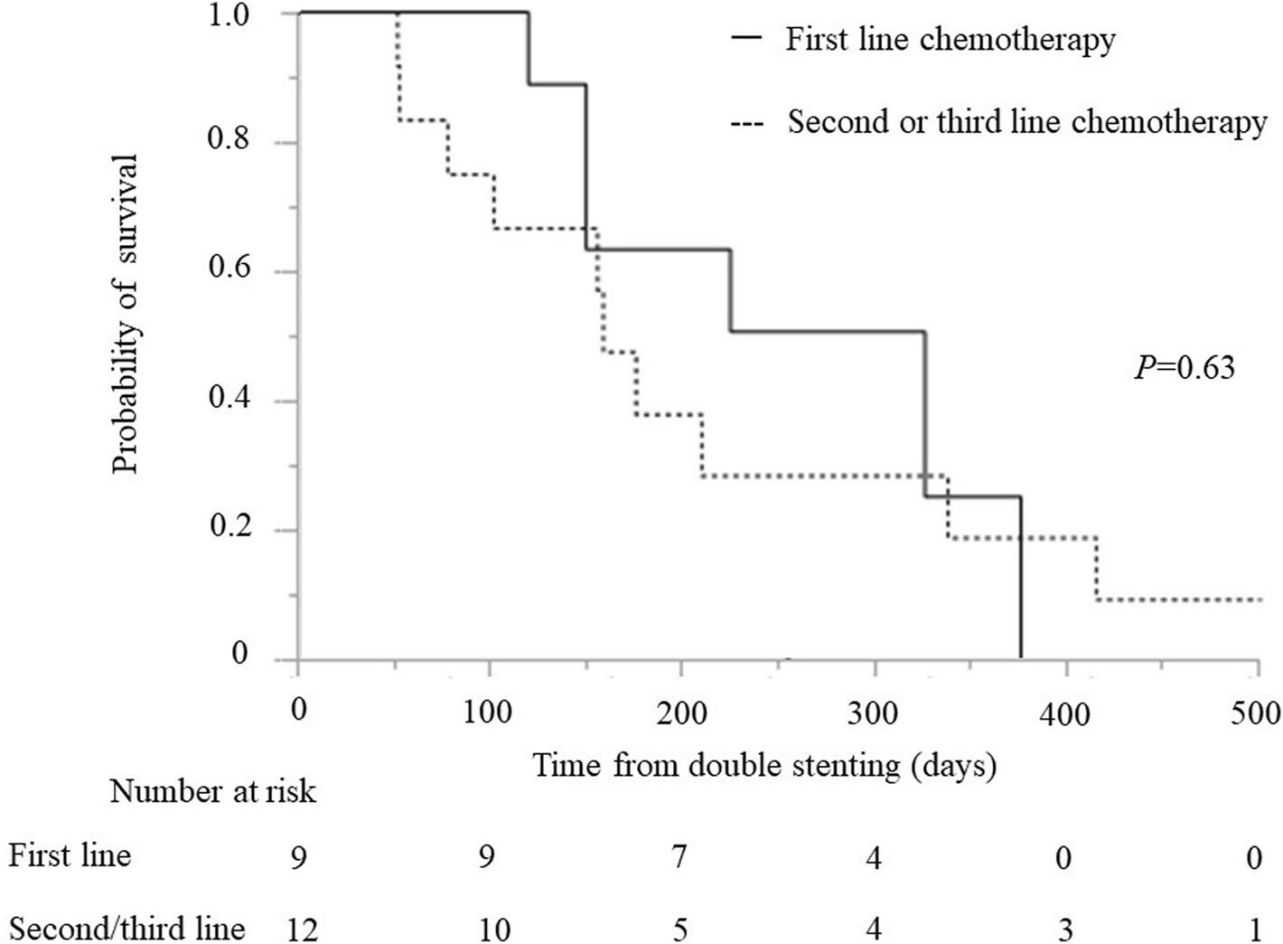
Kaplan-Meier curve showing survival time following double stenting. First line chemotherapy versus second or third line chemotherapy (325 vs 158 days; *P* = 0.63)

Median survival time after double stenting for patients who had any chemotherapy (*n* = 35) and never had any chemotherapy (*n* = 15) was 101 days (IQR 51–224) and 43 days (IQR 34–87), respectively (*P* = 0.0029). Median survival time after double stenting for patients who had upfront double stenting, and then received frontline chemotherapy (*n* = 9) and double stenting during chemotherapy, and then continued second or third line chemotherapy (*n* = 12) was 325 days (IQR 149–375) and 158 days (IQR 89–337), respectively (*P* = 0.63) (Fig. 5).

Median overall survival time for patients who had metastasis (*n* = 33) and without metastasis (*n* = 22) at the initial diagnosis was 264 days (IQR 197–489) and 410 days (IQR 223–791), respectively (*P* = 0.40). Median survival time after double stenting for patients who had metastasis and without metastasis at the initial diagnosis was 53 days (IQR 44–149) and 119 days (IQR 53–175), respectively (*P* = 0.46). Median overall survival time for patients who had simultaneous and metachronous double stenting was 264 days (IQR 123–410) and 367 days (IQR 223–647), respectively (*P* = 0.13). Median survival time after double stenting for patients who had simultaneous and metachronous was 149 days (IQR 49–255) and 53 days (IQR 40–101), respectively (*P* = 0.14).

## Discussion

We retrospectively evaluated the efficacy and safety of post double stenting chemotherapy and analyzed the prognostic factors following double stenting. To our knowledge, this study is the first to evaluate chemotherapy in patients with double stenting for advanced pancreatic cancer.

Recent advances in systemic chemotherapies for pancreatic cancer enable not only first-line, but also second-line and/or third line chemotherapies, even in patients with advanced diseases [17, 18]. Several reports showed the efficacy and safety of systemic chemotherapies for patients with advanced pancreatic cancer with GOO [13, 15]. Kobayashi et al. [15] showed that endoscopic DuS enables the initiation of systemic chemotherapy in more than 50% (36/69) of patients. During treatment, 32% (11/36) of patients developed grade 3–4 anemia, but no fetal adverse events associated with chemotherapy occurred. Survival analysis after post-stenting showed that introduction of chemotherapy was an independent factor of survival [HR: 1.85 (1.02–3.38), *P =* 0.045]. Stephen et al. [13] reported that 50% (98/196) of patients with advanced pancreatic cancer could receive chemotherapy post duodenal stent placement. The median survival periods of the patients with post stent chemotherapy and without chemotherapy were 5.4 and 1.5 months (*P* < .0001), respectively. In our study, 42% (21/50) of patients could receive chemotherapy even after double stenting. Moreover, 12 (46%) out of 26 patients who received chemotherapy before double stenting could continue another chemotherapy, and 9 (64%) out of 14 patients with simultaneous onset at the initial diagnosis could start initial chemotherapy. Median survival time after double stenting for patients who had upfront double stenting, and then received frontline chemotherapy (*n* = 9) and double stenting during chemotherapy, and then continued second or third line chemotherapy (*n* = 12) was 325 days (IQR 149–375) and 158 days (IQR 89–337), respectively (*P* = 0.63). There was no significant difference between front line chemotherapy and second or third line thermotherapy. Thus, we consider that continuing the chemotherapy even if second or third line had improved the survival time after double stenting. During chemotherapy, 8 (38%) patients had grade 3–4 adverse events, which were manageable. Of the 64 patients with advanced pancreatic cancer who had received chemotherapy (first line regimen; GEM 34, S-1 13, mFOLFIRINOX 11, GEM+S-1 6) without double stenting during 2012 October to 2015 October in our institution, 48% (31/64) patients had grade 3–4 adverse events (neutropenia in 22, leukopenia in 10, thrombocytopemia in 2, appetite loss in 3, diarrhea in 2, fatigue in 1, and rush in 1: 10 patients had duplication). For first-line chemotherapy, the incidence of grade 3–4 adverse event was 44% (4/9) in patients with double stenting and 48% (31/64) in patients without double stenting, respectively (*P* = 0.82). Therefore, we considered that there was some tolerability of chemotherapy for the patients with double stenting. For survival analysis, the median survival time after double stenting was significantly longer in patients with chemotherapy post double stenting (175 days) than that in patients without chemotherapy (46 days) (*P* = <.0001). Although the number of patients who received chemotherapy was small, administration of chemotherapy even after double stenting may contribute to prolonged prognosis.

Other prognostic factors after double stenting were reintervention for biliary stent dysfunction and performance status (< 2). The level of performance status is well known prognostic factor of chemotherapy for cancer [15]. In a recent report, the reintervention for biliary and duodenal stent dysfunction is technically feasible and clinically effective even after double stenting [5, 9]. In our study, the clinical success rate for biliary reintervention was 92% (11/12), and one patient who experienced clinical failure following ERCP-BD underwent an additional intervention using EUS-HGS. Out of 12 patients, 9 patients had received chemotherapy at the biliary stent obstruction. After reintervention, 8 patients could continue the chemotherapy. Moreover, in patients with stent dysfunction, the median time of stent dysfunction after double stenting was 87 days (range 25–270 days). The median survival time after double stenting in patients with no biliary stent dysfunction was 53 days (range 17–375) (median was not significant difference, *P* = 0.21). Thus, if the endoscopic reintervention was not performed for biliary dysfunction, the survival time was not prolonged. Appropriate reintervention enabled to restart the chemotherapy, as the reintervention for biliary stent was the independent prognostic factor in this study. On the other hand, duodenal stent dysfunction occurred in only 6 patients. Although all patients who experienced stent dysfunction underwent endoscopic reintervention successfully, it did not show a prognostic value in this study.

This study has 2 limitations. First, it has a retrospective, single center design and comprises a small number of patients who received chemotherapy. Second, the patients did not receive the same resume and the line of chemotherapy after double stenting; various types of MS (covered or uncovered type) or PS were used. Thus, the survival period and stent dysfunction of both the duodenal and biliary stents were difficult to evaluate. These limitations are biased against the prognostic factors.

## Conclusions

In conclusion, systemic chemotherapy was manageable, even in patients with double stenting. The chemotherapy after double stenting and appropriate reintervention for stent obstructions potentially prolonged the survival of patients with advanced pancreatic cancer.

## Abbreviations

BD: Biliary drainage
BS: Biliary stenting
BSC: Best supportive care
CIs: Confidence intervals
CPT-11: Irinotecan
CT: Computed tomography
DuS: Duodenal stenting
ERCP: Endoscopic retrograde cholangiopancreatography
ERCP-BD: Endoscopic retrograde cholangiopancreatography-guided biliary drainage
EUS: Endoscopic ultrasound
EUS-CDS: Endoscopic ultrasound-guide choledochoduodenostomy
EUS-HGS: Endoscopic ultrasound-guided hepaticogastrostomy
GEM: Gemcitabine
GOO: Gastric outlet obstruction
GOOSS: Gastric outlet obstruction scoring system
HR: Hazard ratio
IQR: Interquartile range
mFOLFIRINOX: modified FOLFIRINOX
MS: Metallic stent
ORs: Odd ratios
PS: Plastic stent
S-1: TS-1
SD: Standard deviation
